# Sustainable nanomaterials for precision dental medicine: green synthesis, therapeutic applications, and future directions

**DOI:** 10.1186/s12951-025-04008-3

**Published:** 2026-02-06

**Authors:** Al-Hassan Soliman Wadan, Mohamed Hany Ali, Doha El-Sayed Ellakwa

**Affiliations:** 1 Department of Oral Biology, Faculty of Dentistry, Galala Plateau, Galala University, Attaka, 15888 Suez Egypt; 2 Faculty of Medicine, Helwan University, Helwan, Cairo Governorate Egypt; 3 Department of Biochemistry and Molecular Biology, Faculty of Pharmacy for Girls, Al-Azhar University, Cairo, Egypt; 4 Department of Biochemistry, Faculty of Pharmacy, Sinai University, Kantra Branch, Ismailia, Egypt; 5 Molecular Biology and Genetic Sciences, National Committee for Biochemistry, Academy of Scientific Research and Technology, Cairo, 11516 Egypt

**Keywords:** Sustainable nanomaterials, Precision dental medicine, Green synthesis nanotechnology, Regenerative dentistry, Nano-enabled oral diagnostics

## Abstract

Sustainable nanomaterials are emerging as transformative platforms for precision dental medicine, uniquely combining environmental responsibility with individualized therapeutic performance. Green-synthesized metallic, polymeric, carbon-based, and bioactive nanomaterials exhibit superior biocompatibility, biodegradability, and a reduced ecological burden compared to conventionally produced analogues, while enabling enhanced antimicrobial, regenerative, and diagnostic capabilities. This review synthesizes recent advances in eco-friendly nanoparticle synthesis, life-cycle sustainability metrics, and the integration of nanotechnology into patient-specific diagnostics, controlled-release therapeutics, and regenerative dentistry. Emphasis is placed on biogenic routes for silver, gold, ZnO, chitosan, bioactive glass, cellulose nanocrystals, and lignin nanocarriers, as well as their clinical potential in caries management, periodontal regeneration, endodontic disinfection, implant surface engineering, and point-of-care diagnostics. Additionally, their compatibility with multi-omics-driven precision dentistry is highlighted. We further analyze safety profiles, biodegradation pathways, regulatory frameworks, and translational challenges related to standardization and AI-assisted personalization. Sustainable nano-platforms represent a strategic route to advance dental care toward predictive, preventive, and personalized practice while ensuring environmental stewardship and global healthcare equity.

**Graphical abstract:**

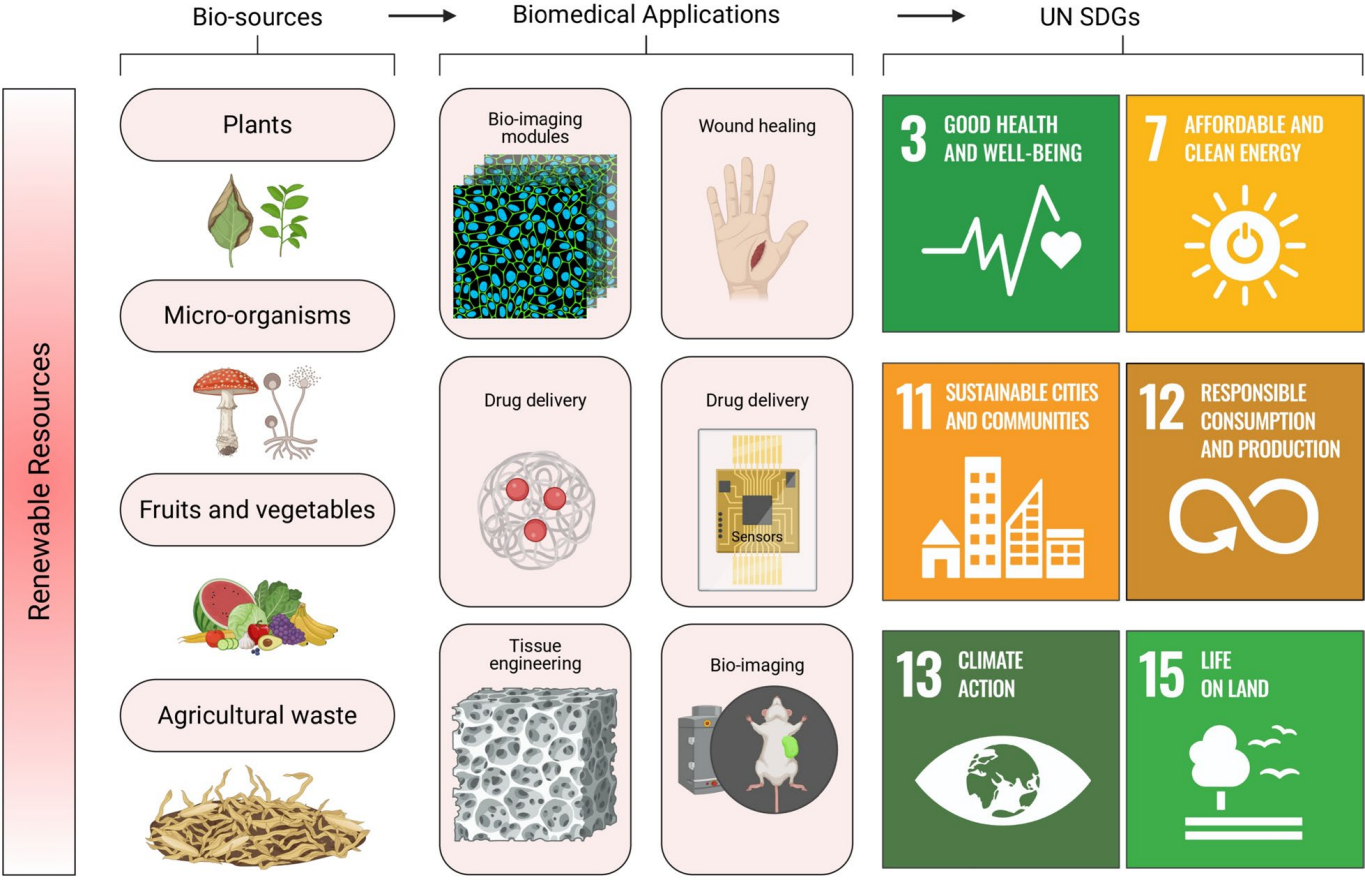

## Introduction

The convergence of sustainability science and precision medicine represents a paradigm shift in modern healthcare, with dental medicine positioned uniquely to benefit from this transformative approach [1]. Sustainable nanomaterials, defined as nanoscale materials synthesized through environmentally benign processes with minimal ecological footprint and enhanced biocompatibility, are emerging as critical components in the development of next-generation dental therapeutics [2–4]. These materials, characterized by their biodegradability, non-toxicity, and renewable synthesis pathways, offer unprecedented opportunities to address the growing environmental concerns associated with conventional nanomaterial production while maintaining superior therapeutic efficacy [5–8]. While their therapeutic potential in dentistry has been recognized, their integration into clinical practice remains limited by challenges related to scalability, reproducibility, and regulatory frameworks.

Precision medicine in dentistry encompasses the customization of healthcare decisions, treatments, and therapeutic products tailored to individual patients based on their genetic profile, environmental factors, lifestyle, and specific oral health conditions [9]. The integration of nanotechnology into precision dental medicine enables unprecedented control over therapeutic delivery, diagnostic sensitivity, and regenerative outcomes through molecular-level interventions [9]. This approach represents a fundamental departure from the traditional "*one-size-fits-all*" methodology, offering personalized solutions that consider the unique biological signatures of each patient’s oral microenvironment.

However, despite the promising laboratory-scale success of green-synthesized nanomaterials, their large-scale production and clinical application are hindered by unresolved tensions between idealized green synthesis methods and the practical, economic, and regulatory demands of industrial-scale manufacturing. This gap represents a critical, unresolved issue in the field, which this review seeks to address by focusing on the challenges and opportunities that exist at the intersection of sustainability and precision in dental nanomedicine.

The urgency for sustainable approaches in dental nanomedicine originates from mounting evidence of environmental toxicity and bioaccumulation associated with conventionally synthesized nanomaterials [10, 11]. Traditional chemical synthesis methods often employ toxic reducing agents, stabilizers, and organic solvents that pose significant ecological risks and potential long-term health consequences [11]. In contrast, green synthesis approaches utilizing biological entities such as plant extracts, microorganisms, and biomolecules offer environmentally responsible alternatives that maintain or enhance therapeutic efficacy while minimizing environmental impact [12–14].

This review synthesizes recent advances in green nanomaterial synthesis and evaluates their applications in dental care, including antimicrobial therapies, diagnostic platforms, and regenerative treatments. It critically examines state-of-the-art green synthesis approaches, their environmental advantages, and their clinical relevance for managing conditions such as periodontal disease and implant-associated infections. Distinct from prior reviews that address isolated aspects of green nanomaterials or nanodentistry, this work provides an integrated perspective on the scalability of sustainable nanomaterials for precision dental medicine, while identifying key gaps in the literature and emerging opportunities to bridge laboratory research and clinical practice, particularly with respect to reproducibility, cost-effectiveness, and regulatory approval.

## Sustainable nanomaterials: principles and green synthesis strategies

Green nanotechnology represents a revolutionary approach to nanomaterial synthesis that prioritizes environmental sustainability, energy efficiency, and biological safety throughout the entire material lifecycle [3]. This paradigm emphasizes the utilization of renewable resources, non-toxic reagents, and energy-efficient processes while minimizing waste generation and environmental contamination [5, 15, 16]. The fundamental principles of green nanotechnology align with the twelve principles of green chemistry, incorporating atom economy, safer solvents, renewable feedstocks, and catalytic efficiency to create nanomaterials with superior environmental profiles [17, 18].

**Fig. 1:**
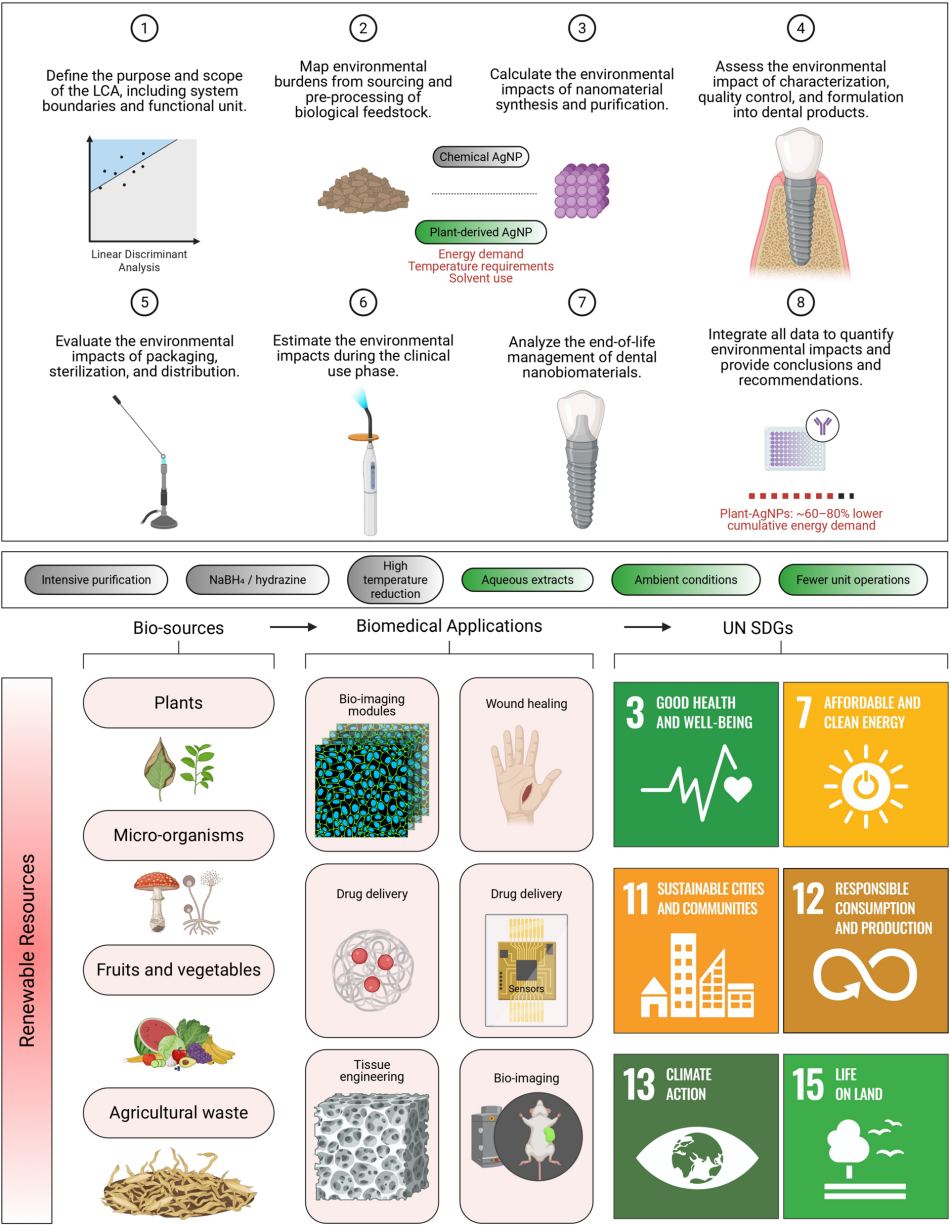
Comparative life-cycle assessment (LCA) framework for sustainable silver nanobiomaterials (AgNPs) in dental applications. The upper panel presents a cradle-to-end-of-life LCA workflow encompassing goal and scope definition, feedstock sourcing and pre-processing, nanomaterial synthesis and purification, formulation into dental products, packaging and distribution, clinical use, and end-of-life management. A focused comparative case study is embedded at the synthesis stage, contrasting conventional chemically synthesized AgNPs with plant-derived AgNPs intended for dental use. Chemical synthesis is characterized by high-temperature reduction, intensive purification, and the use of hazardous reductants (e.g., NaBH₄ or hydrazine). In contrast, plant-mediated synthesis employs aqueous extracts, ambient conditions, and fewer unit operations. This comparison highlights an estimated ~ 60–80% reduction in cumulative energy demand for plant-derived AgNPs relative to chemical routes. The lower panel illustrates renewable biological feedstocks (plants, microorganisms, fruits and vegetables, and agricultural residues), representative biomedical and dental applications (bio-imaging, drug delivery, wound healing, sensing, and tissue engineering), and their alignment with relevant United Nations Sustainable Development Goals (SDGs 3, 7, 11, 12, 13, and 15). All illustrations were created using BioRender.com

Life-cycle assessment (LCA) serves as a critical tool for evaluating the environmental impact of nanomaterial production, providing quantitative metrics for energy consumption, carbon footprint, and ecological toxicity throughout the material’s lifespan (see Fig. 1) [19–22]. Energy efficiency in green synthesis protocols significantly reduces the environmental burden associated with nanomaterial production, with biogenic synthesis routes typically requiring 60–80% less energy compared to conventional chemical methods [23, 24]. The elimination of harsh chemicals, high-temperature processes, and toxic stabilizers further enhances the sustainability profile of green-synthesized nanomaterials.

Green-synthesized metallic nanoparticles represent a cornerstone of sustainable nanotechnology for dental applications. Silver nanoparticles synthesized using plant extracts such as *Azadirachta indica*, *Ocimum sanctum*, and *Camellia sinensis* demonstrate superior antimicrobial activity against oral pathogens while exhibiting reduced cytotoxicity compared to chemically synthesized counterparts [25–29]. Gold nanoparticles produced through biogenic routes using bacterial systems or plant polyphenols maintain excellent biocompatibility and plasmonic properties essential for diagnostic applications [30–34]. Zinc oxide nanoparticles synthesized via green chemistry approaches show enhanced photocatalytic activity and antimicrobial efficacy while demonstrating improved biodegradability profiles [35–37].

Biopolymer nanomaterials derived from renewable sources constitute another significant class of sustainable nanomaterials for dental medicine. Chitosan nanoparticles, extracted from *crustacean shells* or produced through fungal fermentation, offer exceptional mucoadhesive properties, biocompatibility, and controlled release capabilities for oral drug delivery [38–40]. Cellulose nanocrystals and nanofibrils derived from agricultural waste offer structural reinforcement for dental composites while maintaining complete biodegradability [41, 42]. Lignin nanoparticles, sourced from paper industry byproducts, demonstrate antioxidant properties and serve as sustainable platforms for therapeutic delivery in periodontal applications [43–45].

Bioactive glass nanoparticles synthesized through green sol–gel routes utilizing bio-derived silica sources represent innovative approaches to sustainable dental regeneration materials [46–48]. These materials, produced using rice husk ash or bamboo leaf extracts as silica sources, maintain superior bioactivity and osteoconductivity while reducing dependence on traditional chemical precursors. The incorporation of natural calcium and phosphorus sources further enhances the sustainability profile of these materials while promoting biomineralization processes essential for dental tissue regeneration [49].

**Fig. 2:**
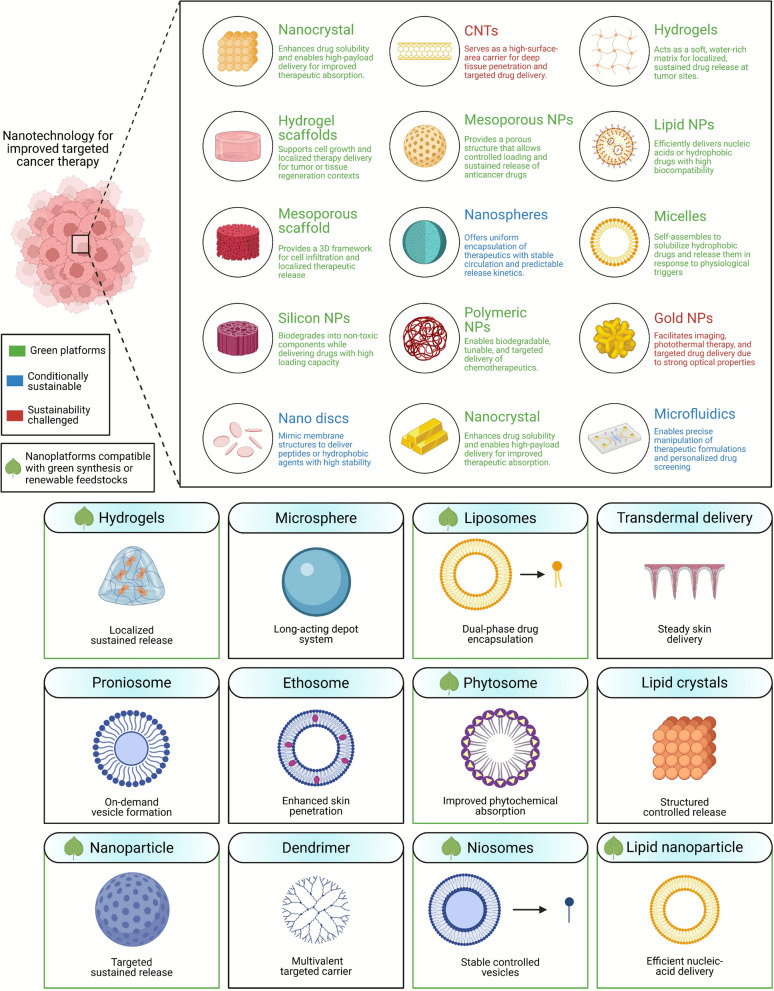
Sustainability-annotated nanoplatforms and delivery systems for precision biomedical applications. The upper panel presents a representative spectrum of nanoscale therapeutic platforms, including nanocrystals, hydrogels, mesoporous architectures, silicon-based nanoparticles, polymeric systems, lipid-based carriers, micelles, nanodiscs, gold nanoparticles, carbon nanotubes, and microfluidic assemblies. Platforms are visually classified according to their sustainability potential. The nanoplatforms compatible with green synthesis routes or renewable feedstocks are highlighted (green), conditionally sustainable systems are indicated (blue), and platforms associated with higher material or energy burdens are denoted as sustainability-challenged (red). This annotation links structural and functional attributes, such as drug-loading modality, degradation behavior, membrane mimicry, and release kinetics, to environmentally informed design choices. The lower panel illustrates advanced delivery configurations, including vesicular carriers, particulate depots, transdermal systems, phytoconjugated assemblies, dendritic architectures, and lipid nanoparticles. Selected delivery systems derived from bio-based components or amenable to low-energy fabrication routes are marked accordingly, emphasizing how platform selection can directly influence the sustainability profile of biomedical and dental nanotechnologies

There are many nanoengineered platforms used in targeted therapy and advanced drug-delivery systems (see Fig. 2). Sustainability metrics for evaluating green nanomaterials encompass multiple parameters, including biodegradability rates, energy footprint calculations, renewable content percentages, and ecotoxicity assessments [2, 49–53]. Biodegradability testing using standardized protocols demonstrates that green-synthesized nanomaterials typically degrade 3–5 times faster than conventional alternatives, reducing long-term environmental accumulation [54–58]. Energy footprint analyses reveal that biogenic synthesis routes can achieve up to an 85% reduction in energy consumption compared to traditional high-temperature synthesis methods. Renewable content assessments quantify the proportion of bio-derived components in nanomaterial formulations, with sustainable alternatives achieving 70–95% renewable content compared to 10–20% for conventional materials [59–61]. These comprehensive sustainability metrics provide essential frameworks for evaluating and optimizing green nanomaterial synthesis protocols, ensuring both therapeutic efficacy and environmental responsibility in dental medicine applications.

While green synthesis approaches are often justified based on reduced cytotoxicity and environmental burden, their actual mechanistic advantages extend from the unique biomolecular coronas imparted by biogenic reducing and stabilizing agents. Plant- and microbe-derived extracts introduce complex surface chemistries enriched in polyphenols [62, 63], flavonoids [64, 65], polysaccharides [66, 67], proteins [68], and organic acids [68], which act not only as stabilizing capping layers but also as functional biointerfaces that modulate nanoparticle behavior in the oral microenvironment. These biomolecular coatings enhance colloidal stability under dynamic salivary conditions by providing steric and electrostatic stabilization across fluctuating pH, ionic strength, and enzymatic activity, thereby reducing aggregation and uncontrolled ion release compared with chemically capped counterparts [69–72]. Significantly, such coronas influence biological identity by shaping protein adsorption patterns from saliva, potentially biasing immune recognition toward tolerogenic or anti-inflammatory responses rather than nonspecific phagocytic clearance. In periodontal and peri-implant niches, biogenic surface functionalities may further facilitate selective interactions with inflamed tissues or dysbiotic biofilms through hydrogen bonding, lectin-like interactions, or redox-responsive behavior, offering a level of microenvironmental responsiveness that is difficult to achieve with inert synthetic ligands. However, these advantages are intrinsically coupled to compositional heterogeneity and batch variability, underscoring a critical trade-off between biological functionality and regulatory reproducibility [69–72].

## Precision medicine in dentistry: concepts and clinical needs

Precision dentistry represents a transformative approach to oral healthcare that integrates individual patient characteristics, genetic predispositions, environmental factors, and lifestyle variables to deliver personalized diagnostic and therapeutic interventions [73–75]. This paradigm shift moves beyond traditional symptom-based treatment protocols toward predictive, preventive, and customized care strategies that address the root causes of oral diseases while optimizing treatment outcomes for individual patients. The foundation of precision dentistry rests on comprehensive patient profiling encompassing genomic analysis, proteomic screening, metabolomic assessment, and microbiome characterization to create detailed biological signatures that guide clinical decision-making [76, 77].

Patient-specific diagnosis in precision dentistry leverages advanced molecular techniques to identify disease susceptibility markers, pathogen-specific biomarkers [78], and treatment response predictors that enable early intervention and personalized therapeutic selection [76]. Genetic polymorphisms affecting enamel formation, immune responses, and drug metabolism provide crucial information for customizing preventive strategies and therapeutic protocols [79]. Salivary biomarker analysis enables non-invasive monitoring of inflammatory processes, oxidative stress, and microbial dysbiosis, providing real-time insights into oral health status and treatment efficacy [80–82].

Oral biofilm profiling represents a critical component of precision dentistry, utilizing advanced sequencing technologies and machine learning algorithms to characterize the complex microbial communities inhabiting different oral niches [83–87]. The unique composition and functional characteristics of individual patient microbiomes influence disease susceptibility, progression patterns, and therapeutic responses, necessitating personalized antimicrobial strategies [82]. Metagenomic analysis reveals strain-specific antibiotic resistance patterns, virulence factors, and metabolic pathways that inform targeted therapeutic interventions while preserving beneficial microbial populations [82].

To address the spatial and biological constraints inherent to dental tissues, nanoscale delivery systems have emerged as key enablers of precision dentistry by allowing localized, stimulus-responsive, and patient-tailored therapeutic release. Nanotechnology serves as a critical enabling platform for precision dentistry by providing molecular-level control over therapeutic delivery, diagnostic sensitivity, and tissue engineering approaches. Nanocarriers can be engineered with specific targeting ligands, responsive release mechanisms, and therapeutic payloads tailored to individual patient profiles and disease characteristics. Surface functionalization of nanoparticles with patient-specific antibodies, aptamers, or peptides enables selective targeting of pathogenic bacteria such as Staph. Arues in infected diabetic wounds [88], cancer cells [89–93], or damaged tissues while minimizing off-target effects [94]. In dentistry, precision medicine extends beyond molecular targeting to encompass site-specific anatomy, localized disease microenvironments, and patient-specific functional demands [74]. Dental tissues present unique barriers, including limited vascularization, dynamic mechanical loading, fluctuating pH, and a complex oral microbiome [95], that constrain the effectiveness of conventional systemic or bulk delivery approaches. As a result, precision in dentistry requires therapeutic systems capable of localized, temporally controlled, and microenvironment-responsive intervention. It is within this framework that nanoscale delivery systems become functionally relevant, not as generic nanomedicine platforms, but as enabling technologies that translate precision principles into clinically actionable dental therapies [92].

## Nanosystems for controlled release

Controlled release nanosystems offer unprecedented precision in therapeutic delivery, enabling temporal and spatial control over drug concentrations based on individual pharmacokinetic profiles and disease progression patterns [96–100]. Stimuli-responsive nanocarriers can be designed to release therapeutic agents in response to specific pH conditions, enzymatic activity, or pathogen-associated molecular patterns characteristic of individual patient disease states [94, 101, 102]. This level of control minimizes adverse effects while maximizing therapeutic efficacy through personalized dosing regimens and targeted delivery approaches [103–106]. Regenerative nanotechnology enables precision tissue engineering through the development of patient-specific scaffolds, growth factor delivery systems, and stem cell guidance platforms [100, 107–111]. Nanoscale topographical features, biochemical gradients, and mechanical properties can be tailored to match individual patient anatomy and regenerative capacity, optimizing tissue repair outcomes. The integration of patient-derived stem cells with nanoengineered scaffolds provides personalized regenerative solutions that address the unique biological requirements of each clinical situation [112].

### Bone regeneration and tissue engineering

Bone tissue engineering relies on a triad of scaffold, cells, and bioactive signals [113]. Recent work has exploited biomimetic nanomaterials to enhance each element of this triad. For example, hydroxyapatite (HA) nanoparticles, which are chemically similar to natural bone mineral, can carry osteogenic agents. APTES-modified HA NPs were shown to deliver miR-302a-3p into human osteoblasts, downregulating the COUP-TFII inhibitor and upregulating RUNX2, thereby boosting bone-forming activity [113]. In a mouse cranial defect model, a 3D‐printed tricalcium phosphate/HA scaffold modified with these HA‐miRNA nanocarriers produced significantly more new bone (higher bone volume fraction) and faster defect filling than an unmodified scaffold [114]. Such CaP scaffolds are inherently biocompatible and resorbable, offering an eco-friendly alternative to metal implants. Beyond simple scaffolds, innovative delivery systems have been devised to address metabolic challenges: a glucose‐responsive hydrogel was engineered to release stem-cell‐derived exosomes (rich in *Smpd3* protein) together with nanosilver ions under hyperglycemic conditions, rescuing bone healing in diabetic models. Importantly, these mesenchymal stem cell exosomes are natural nanovesicles (~ 30–150 nm) carrying proteins and RNAs [115]; they exhibit anti-inflammatory targeting, low immunogenicity, and high stability compared to synthetic carriers. Other researchers have been investigating the role of molybdenum-containing bioactive glass ceramic (Mo-BGC) scaffolds in material immunomodulation and periodontal wound healing by targeting immunometabolism and mitochondrial function for macrophage modulation [116].

### Antimicrobial and antifungal therapies

Nanomaterials are also advancing sustainable antimicrobial strategies in dentistry [11, 29, 98, 117–137]. Multifunctional liposomes, for instance, have been used to co-deliver antibiotics and natural photosensitizers. One study loaded *doxycycline* and plant-derived curcumin into nanoliposomes for photodynamic therapy against *A. actinomycetemcomitans* [131]. This hybrid system achieved ~ 82% reduction in pathogenic biofilms and suppressed bacterial metabolism, with minimal harm to host cells [131]. Similarly, herbal extracts have been formulated as nanoparticles [138]. For instance, neem (*Azadirachta*) oil, known for its antibacterial compounds, was tested as nanoparticles alongside calcium hydroxide nanoparticles against root canal pathogens [126]. Low-dose neem NPs inhibited > 59% of *Streptococcus* biofilm and outperformed bulk neem oil, while Ca(OH)₂ NPs killed *S. mutans* and *E. faecalis* more effectively than conventional Ca(OH)₂ pastes [126]. Such green nanopreparations harness natural antimicrobials in nanocarrier form to enhance efficacy. For fungal threats, targeted nanocarriers enable precision gene therapy using polymeric PEG–poly(amino acid) micelles, which were engineered to deliver microRNA into *Aspergillus fumigatus*, silencing the alb1 melanin gene [139]. The miRNA-loaded nanomicelles reduced Alb1 protein and melanin, sensitising the fungus to oxidative killing and neutrophils [139]. Ultimately, biodegradable polymer nanoparticles can enhance the efficacy of existing drugs. Ciprofloxacin was encapsulated in FDA-approved PEG–PLGA NPs for root-canal delivery [140]. The 120 nm PEG–PLGA NPs released *ciprofloxacin* over 7 days, significantly enhancing *E. faecalis* killing while sparing dental stem cells [140].

Although antimicrobial nanosystems are frequently positioned as resistance-mitigating alternatives to conventional antibiotics, their behavior within the oral ecosystem reveals important conceptual and translational uncertainties. Unlike antibiotics, which exert selective pressure through defined molecular targets, nanoparticles act via multifactorial physicochemical mechanisms, membrane perturbation, oxidative stress induction, metal ion release, and metabolic interference, which are often assumed to preclude resistance development. However, emerging evidence indicates that prolonged or sub-lethal nanoparticle exposure can drive adaptive microbial responses distinct from classical antimicrobial resistance, including enhanced extracellular matrix production, nanoparticle sequestration, altered membrane composition, and stress-response reprogramming that collectively reduce nanoparticle efficacy without requiring genetic target mutations [141]. This concern is particularly salient in dentistry, where nanomaterials may persist chronically within restorative materials, implant coatings, or periodontal niches, creating sustained selective pressures within structured multispecies biofilms. Moreover, most antimicrobial nanoparticles lack true pathogen specificity, relying instead on non-discriminatory mechanisms such as ROS generation or membrane disruption, which risks collateral damage to commensal oral microbiota essential for ecological stability and host defense; such disruption may paradoxically exacerbate dysbiosis, facilitating recolonization by more resilient or opportunistic taxa rather than restoring microbial homeostasis. The challenge is further compounded for green-synthesized nanoparticles, whose biologically derived surface coronas can enhance biocompatibility and sustainability but introduce variability in surface chemistry, ion release kinetics, and microbial interactions, obscuring whether antimicrobial activity arises from the nanoparticle core, released species, or residual phytochemicals [141].

### Diagnostic and theranostic innovations

In dentistry, nanomaterials are increasingly enabling integrated diagnostics and therapy, known as theranostics, allowing for the emergence of innovative approaches in dental research. Trends in dental theranostic development, with gold [11, 30–34, 99, 124, 135, 142–168], iron-oxide [144, 169–178], and carbon-based platforms [179–184] dominating current research due to their multifunctionality and imaging compatibility. Notably, only a subset of these systems, particularly biogenic gold nanoparticles, iron-oxide nanoparticles synthesized via plant or microbial routes, carbon quantum dots, and chitosan- or protein-based nanocarriers, have been explicitly realized using green or bio-assisted synthesis strategies. In contrast, many advanced hybrid and multimodal platforms prioritize performance optimization without reporting sustainability metrics or environmentally benign fabrication routes. Key gaps include inconsistent disclosure of green-synthesis methodologies, limited life-cycle and ecotoxicity assessments, and scarce data on scalable, GMP-compliant production (see Table 1). Such lipid-based nanocarriers, known as liposomes, can be adapted as imaging probes for the oral vasculature or targeted inflammation via MRI. For example, *Gadolinium*-chelating liposomes were developed as MRI contrast agents that can also carry therapeutics [185]. In vitro safety studies demonstrated that these Gd-liposomes caused no toxicity in human hepatocytes or macrophages, thereby affirming their biocompatibility and paving the way for further research in dental applications. Other studies have utilised biomaterial scaffolds with the potential for M2 macrophage polarization [116]. Crucially, these liposomal platforms can co-encapsulate imaging and drug payloads (e.g., a contrast agent with a thrombolytic), thus serving both for the visualization and treatment of disease. This illustrates a broader vision of nanoscale theranostics for dentistry, including the MRI visualization of inflammatory conditions [185].

**Table 1:** Theranostic nanomaterials in dentistry

No	Theranostic platform	Nanomaterial type	Key function(s)	Main application	References
1	SERS–Gold Nanoparticles	Gold NP	Raman detection	OSCC biomarker detection	[151]
2	Anti-EGFR Gold Nanorods	Gold nanorods	Photothermal therapy	OSCC ablation	[147, 186, 187]
3	Hollow Gold Nanospheres	Gold nanoshells	Drug delivery + PTT	Chemo-PTT	[99]
4	DRS Enhanced by AuNPs	Gold NP	Optical enhancement	Non-invasive tumor detection	[99, 100, 107–111, 144, 146, 148, 150, 152–154, 188–194]
5	Folic Acid–IONPs	Iron oxide	T2 MRI imaging	Tumor localization	[170, 195]
6	HA–Magnetic NPs	Iron oxide	siRNA delivery	Gene therapy	[169, 172]
7	αvβ6-SPIONs	Iron oxide	Magnetic hyperthermia	OSCC apoptosis	[178, 196, 197]
8	Iron Carbide NPs	Iron carbide	MRI contrast	Hypoxic tumor imaging	[170, 177]
9	NIR Quantum Dots	Quantum dots	Deep tissue imaging	OSCC fluorescence	[198, 199]
10	Exosome-QD Probe	Quantum dots	Exosome detection	Liquid biopsy	[200]
11	Carbon Quantum Dots	Carbon QDs	Fluorescence + ROS control	Cancer imaging	[201]
12	Folate-Chitosan PLGA	Polymer NP	MRI + OCT	OSCC detection	[202]
13	PDA-Hyperbranched NPs	Polymer NP	PTT + chemo	Synergistic therapy	[201]
14	GRPR–NGO Nanoprobes	Graphene oxide	Infrared imaging	OSCC imaging	[203]
15	Evodiamine–ICG Liposomes	Lipid NP	PDT + chemo	OSCC therapy	[204]
16	Folate–Liposomal Drugs	Lipid NP	Anti-angiogenesis	Targeted delivery	[205]
17	PET-Guided Lipid NPs	Lipid NP	PET imaging	Tumor imaging	[206–208]
18	Mesoporous Carbon NPs	Carbon NP	PTT + PDT	Phototherapy	[209–211]
19	AIE-BSA Nanoparticles	Protein NP	Light PDT	Tumor-specific therapy	[209]
20	Cancer-Cell Membrane NPs	Biomimetic NP	Homologous targeting	PTT/PDT	[209]
21	Gold–Magnetic Hybrid	Hybrid NP	SERS + MRI	CTC detection	[209–211]
22	QD–Silica–Gold Hybrid	Hybrid NP	Imaging + PTT	Multimodal therapy	[143]
23	Fe3O4@Au/rGO	Hybrid NP	Hyperthermia + PTT	Tri-modal therapy	[212]
24	PAMAM Dendrimers	Dendrimers	PET/MRI/FL	Tumor visualization	[213]
25	pH-Responsive AuNPs	Gold NP	pH-triggered release	TME-targeting	[214]
26	Enzyme-Responsive HA NPs	Polymer NP	Enzyme-triggered release	Penetration therapy	[215]
27	Redox-Responsive Disulfide NPs	Polymer NP	GSH-triggered release	Precision therapy	[216]
28	Checkpoint-Loaded NPs	Immune NP	PD-L1 + PTT	Immuno-theranostics	[216–219]
29	CpG–Gold Vaccine NPs	Gold NP	Immune activation	Vaccine theranostics	[163]
30	Macrophage-Reprogramming NPs	Immune NP	M1 polarization	Immune-guided therapy	[220]

## Sustainable nanomaterials for precision oral diagnostics

Precision oral diagnostics powered by sustainable nanomaterials represents a revolutionary advancement in early disease detection and patient-specific monitoring capabilities. There is a clear trend toward plant-mediated green synthesis as the dominant sustainable route for dental nanomaterials, with antimicrobial and antibiofilm applications forming the primary focus. Medicinal plants rich in polyphenols, flavonoids, and terpenoids, most notably *Azadirachta indica* (neem), *Camellia sinensis* (green tea), *Curcuma longa*, *Aloe vera*, *Punica granatum*, and *Syzygium aromaticum*, merge as the most frequently utilized sources. Their prevalence reflects both their substantial reducing and stabilizing capacity during nanoparticle formation and their intrinsic antimicrobial, anti-inflammatory, and antioxidant properties, which directly support caries prevention, periodontal therapy, endodontic disinfection, and enhancement of restorative materials [221, 222]. Silver-based systems dominate across these applications, highlighting a pattern in which sustainability efforts are preferentially applied to well-established antimicrobial platforms rather than to more complex therapeutic modalities.

Beyond infection control, a secondary but growing trend is the use of plant-derived nanomaterials for regenerative and functional applications, including enamel remineralization, osseointegration, pulp preservation, and modification of implant surfaces. However, these examples remain comparatively limited and are primarily confined to gold, iron oxide, calcium-based, and hydroxyapatite systems derived from a narrow range of botanical or biowaste sources. Notable gaps persist in the sustainable synthesis of advanced diagnostic, theranostic, and immunomodulatory nanoplatforms, as well as in the development of standardized, scalable green protocols supported by long-term biocompatibility, toxicity, and life-cycle assessments. Collectively, these trends suggest that while green nanotechnology in dentistry is well-established for antimicrobial purposes, its extension to multifunctional and clinically translatable platforms remains an essential and underexplored future direction (see Table 2). These innovative biosensing platforms combine the environmental benefits of green-synthesised nanomaterials with the precision and sensitivity required for molecular-level diagnostic applications [223]. The integration of sustainable nanotechnology with advanced detection methodologies enables the development of point-of-care diagnostic systems that provide real-time [224], accurate, and environmentally responsible solutions for oral healthcare monitoring.

**Table 2:** A comprehensive table of different types of nanomaterials, their sources, and their applications in dentistry

Serial	Nanoparticle type	Source	Application	Reference
**Silver nanoparticles (AgNPs)**				
1	AgNP	*Acacia senegal*	Vigorous antibiotic activity against *S. mutans*	[225–227]
2	AgNP	*Camellia sinensis (green tea)*	Vigorous antibacterial activity against *S. mutans*; biofilm inhibition	[132, 226]
3	AgNP	*Azadirachta indica and Aloe vera*	Effective against S. *mutans* and *Pseudomonas species*	[228, 229]
4	AgNP	*Viola serpens*	Effective against strains of *S. mutans*	[230]
5	AgNP	*Curcuma aromatica*	Antibacterial action; biofilm inhibition; microbicidal activity in PMMA films	[231]
6	AgNP	*Oryza sativa L. (rice)*	Vigorous antibacterial activity against *S. mutans*; biofilm inhibition	[232, 233]
7	AgNP	*Punica granatum L. (pomegranate)*	Vigorous activity against *C. albicans* and *S. mutans*	[234, 235]
8	Ag_2_ONP	*Ficus benghalensis*	Active against *S. mutans* and* Lactobacillus suppurative*	[120]
9	AgNP	*Aloe vera*	Antibacterial activity in intracanal medications	[98, 121]
10	AgNP	*Mangifera indica*	AgNP-reinforced GIC; antibacterial activity	[236]
11	AgNP	*Prunus japonica*	Highest activity against *Proteus vulgaris*	[237]
12	AgNP	*Psoralea corylifolia*	Potential in various disorders	[125, 238]
13	AgNP	*Azadirachta indica*	Both Gram-positive and Gram-negative pathogens were susceptible to AgNPs	[128, 129, 228]
14	AgNP	*Salix alba*	Antibacterial against dental plaque bacteria	[239]
15	AgNP	*Emblica officinalis*	Bioreductant for AgNP production	[222]
16	AgNP	*Justicia glauca*	Antibacterial and antifungal activities; MIC of 25–75 µg/mL against tested bacteria	[240]
17	AgNP	*Eucalyptus oleosa*	Effective antibacterial actions	[122]
18	AgNP	*Moringa oleifera*	Effective antibacterial activity against common endodontic bacteria; potential for irrigation solutions	[241]
19	AgNP	*Ocimum sanctum (Tulsi)*	Same optical and antibacterial properties; biomolecules (quercetin) in tulsi largely responsible for reducing metal ions to MNPs	[118, 242]
20	AgNP	*Syzygium aromaticum (clove)*	Enhanced antibacterial properties in adhesive formulations; maintains bond strength	[117, 123, 243]
21	AgNP	*Terminalia chebula*	Reduced plaque formation; safe for oral tissues; natural alternative to synthetic antimicrobials	[244]
22	AgNP	*Psidium guajava (guava)*	Antifungal activity against Candida species	[245]
**Gold nanoparticles (AuNPs)**				
23	AuNP	*Anogeissus latifolia*	Effective analgesic and bone-inducing agents in implantation therapy	[166, 246]
24	AuNP	*Salacia chinensis*	Can be used as efficient bone inductive agent during dental implant therapy; stable, biocompatible, environmentally friendly	[247–249]
25	AuNP	*Indigofera tinctoria*	Cell viability declines with increased nanoparticles; more damaging to cancer cells than pure leaf extract; strong antibacterial activity; higher antioxidant activity	[156, 158, 167, 250, 251]
26	AgNP	*Indigofera tinctoria*	Strong antibacterial activity; enhanced antioxidant properties	[250, 252]
27	AuNP	*Alternanthera philoxeroides*	Successful production; enhancement in gold nanoparticle antibacterial efficacy	[157, 165, 253–255]
28	AuNP	*Justicia glauca*	Potential antibacterial and antifungal action against oral infections; MIC values of 6.25–25 µg/mL	[135, 159, 162, 164, 256]
29	AuNP	*Panax ginseng*	Biosynthesized AuNP characterized; antibacterial properties demonstrated	[161, 257, 258]
30	AgNP	*Panax ginseng*	Antibacterial properties	[161, 257, 258]
31	AuNP	*Stevia rebaudiana*	Leaf extract can produce gold nanoparticles successfully	[259, 260]
32	AuNP	*Curcuma longa (turmeric)*	Enhanced fibroblast proliferation; anti-inflammatory effects; promotes healing of periodontal defects	[133, 149, 261]
33	AuNP	*Camellia sinensis*	Highly sensitive detection of oral cancer biomarkers; early diagnosis potential	[262, 263]
34	AuNP	*Glycyrrhiza glabra (licorice)*	Antibacterial activity against E. faecalis; potential irrigation adjunct	[124, 168, 264]
**Copper nanoparticles (CuNPs/CuO)**				
35	CuNP	*Celastrus paniculatus*	Used as photocatalysts and antifungal agents	[265]
36	CuNP	*Cardiospermum halicacabum*	Inhibits biofilm formation by adhering to cell wall and disrupting growth and development	[265–267]
37	CuNP	*Zingiber officinale*	Free radical scavenging activity	[268]
38	CuNP	*Eryngium caucasicum*	Clean, simple, cost-effective, and efficient technique for green copper nanoparticle production	[269]
39	CuNP	*Plectranthus amboinicus*	Free of contaminants	[270]
40	CuO NP	*Madhuca longifolia*	Strong antibacterial action against *E. coli*, *S. aureus*, and *B. subtilis*; comparable to ampicillin and tetracycline	[271]
41	CuNP	*Azadirachta indica*	Potential for large-scale production	[272]
42	CuNP	*Punica granatum*	Effective antibacterial agents against Staphylococcus aureus	[273, 274]
43	CuNP	*Eclipta prostrata*	Impressive antioxidant potential	[275, 276]
44	CuNP	*Citrus medica Linn*	Considerable inhibitory efficacy against tested microorganisms	[277, 278]
45	CuNP	*Allium sativum (garlic)*	Antibacterial coating for titanium implants; prevents bacterial adhesion	[279, 280]
46	CuO NP	*Musa paradisiaca (banana peel)*	Effective against acid-producing bacteria; natural waste utilization	[281]
**Iron nanoparticles (FeNPs/Fe**_**3**_**O**_**4**_**)**				
47	Fe_3_O_4_ NPs	*Euphorbia hirta*	Highly encouraging antibacterial and antifungal results against various bacterial and fungal pathogens	[282, 283]
48	FeNP	*Rose, Azadirachta indica (neem), carom, Syzygium aromaticum (clove)*	Crystalline structure maintained 3–4 months with PVP presence	[248]
49	FeONP	*Moringa oleifera*	Reusable three times in fluoride ion adsorption; potential material for fluoride ion removal; shortest contact time to equilibrium	[175, 284]
50	FeNP	*Syzygium aromaticum (clove), Azadirachta indica (neem), Camellia sinensis (green tea)*	Adding FeNP to antibacterial treatment boosts its action	[248, 285]
51	FeNP	*Phyllanthus emblica*	Promotes enamel remineralization; biomimetic approach	[286, 287]
52	Fe_3_O_4_ NPs	*Hibiscus rosa-sinensis*	Magnetic-guided delivery system; controlled release of antimicrobials	[173, 174, 286]
**Titanium dioxide nanoparticles (TiO**_**2**_**)**				
53	TiO_2_ NP	*Azadirachta indica twigs, Ficus benghalensis, Syzygium aromaticum*	Outstanding antibacterial and antibiofilm characteristics against *S. mutans*, *C. freundii*, and *C. albicans*	[288]
54	TiO_2_ NP	*Citrus aurantifolia*	Can be used as excellent fillers for light-curing dental nanohybrid composites; improved physical characteristics; antibacterial, hydrophilic, self-cleaning capabilities	[289–291]
55	TiO_2_ NP	*Echinacea purpurea*	Existence of TiO₂ nanoparticles established using UV–Vis, FTIR, and TXRF	[292, 293]
56	TiO_2_ NP	*Acanthophyllum laxiusculum*	TiO₂ nanospheres determined using UV–Vis absorption spectra and confirmed using diffuse reflectance spectroscopy	[294, 295]
57	TiO_2_ NP	*Mentha arvensis*	Promising antibacterial and antifungal action against oral pathogens	[296]
58	TiO_2_ NP	*Aloe barbadensis*	Light-activated whitening with minimal sensitivity; cosmetic dentistry application	[297]
59	TiO_2_ NP	*Vaccinium myrtillus (blueberry)*	Enhanced osseointegration; improved biocompatibility	[298]
**Zinc oxide nanoparticles (ZnO/ZnNPs)**				
60	ZnONP	*Dysphania ambrosioides*	Most bacterial strains susceptible to synthetic and commercial NPs; Prevotella intermedia most sensitive to ZnONPs	[299, 300]
61	ZnONP	*Sesamum indicum L*	Cost-effective ZnONP synthesis with potential for further exploration	[301]
62	ZnONP	*Juglans regia L*	Green approach ZnO nanoparticles had stronger antibacterial impact; lower cytotoxicity than chemically synthesized	[221, 299, 301]
63	ZnONP	*Salvadora persica*	UV–Vis investigations indicated ZnO nanoparticle production; toxicity proportional to nanoparticle concentration	[302]
64	ZnNP	*Lavandula vera*	Changes in oxidative stress unrelated to caspase pathway; NOAEL < 1 g/kg in 14-day subacute toxicity trial	[303]
65	ZnONP	*Costus pictus D. Don*	Good antibacterial efficacy against bacterial and fungal species; anticancer effect against DLA cells	[304]
66	ZnONP	*Citrus limon (lemon)*	Enhanced mechanical properties; improved flexural strength	[305]
67	ZnONP	*Cinnamomum verum (cinnamon)*	Improved antibacterial sealing ability; enhanced root canal treatment	[305]
68	ZnONP	*Origanum vulgare (oregano)*	Reduced inflammation; antibacterial properties; natural therapeutic agent	[305, 306]
**Platinum nanoparticles (PtNPs)**				
69	PtNP	*Punica granatum*	Free radical scavenging properties; enamel protection; oxidative stress reduction	[307]
70	PtNP	*Camellia sinensis*	Sensitive detection of inflammatory cytokines; an early diagnosis tool	[308, 309]
**Palladium nanoparticles (PdNPs)**				
71	PdNP	*Gardenia jasminoides*	Enhanced hydrogen peroxide activation; improved whitening efficiency	[308, 310]
72	PdNP	*Cinnamomum camphora*	Broad-spectrum antibacterial activity; maintains adhesive properties	[311]
**Selenium nanoparticles (SeNPs)**				
73	SeNP	*Allium sativum (garlic)*	Inhibits S. mutans biofilm formation; low toxicity to oral tissues	[312, 313]
74	SeNP	*Emblica officinalis*	Reduces oxidative stress in gingival tissues; anti-inflammatory effects	[314, 315]
75	SeNP	*Vitis vinifera (grape seed)*	Promotes fibroblast proliferation; enhances wound healing	[316]
**Cerium oxide nanoparticles (CeO**_**2**_**)**				
76	CeO_2_ NP	*Aloe vera*	Promotes dentin bridge formation; pulp tissue preservation	[317]
77	CeO_2_ NP	*Moringa oleifera*	Reduces pro-inflammatory cytokines; periodontal therapy	[318]
78	CeO_2_ NP	*Hibiscus sabdariffa*	Mimics SOD enzyme activity; protects against oxidative damage	[319]
**Magnesium oxide nanoparticles (MgO)**				
79	MgONP	*Syzygium aromaticum*	Prevents white spot lesions during orthodontic treatment	[320]
80	MgONP	*Ocimum sanctum*	Enhanced fluoride release; antibacterial properties; improved mechanical strength	[321]
**Calcium carbonate nanoparticles (CaCO**_**3**_**)**				
81	CaCO_3_ NP	*Eggshell* + *Aloe vera*	Biomimetic hydroxyapatite formation; natural remineralization	[322]
82	CaCO_3_ NP	*Sepia officinalis (cuttlefish bone)*	Osteoconductive scaffold; bone regeneration support	[323]
**Hydroxyapatite nanoparticles (nHA)**				
83	nHA	*Seashell* + *Moringa oleifera*	Enhanced biocompatibility; improved osseointegration	[324–326]
84	nHA	*Eggshell* + *Curcuma longa*	Occludes dentinal tubules effectively; reduces hypersensitivity	[327, 328]
85	nHA	*Oyster shell* + *Camellia sinensis*	Restores enamel microhardness; prevents demineralization	[329]
**Silica nanoparticles (SiO**_**2**_**)**				
86	SiO_2_ NP	*Rice husk* + *Azadirachta indica*	Improved mechanical strength; natural waste valorization	[330, 331]
87	SiO_2_ NP	*Bamboo leaf ash* + *Aloe vera*	Enhanced surface smoothness; biocompatible polishing agent	[332, 333]
**Manganese oxide nanoparticles (MnO**_**2**_**)**				
88	MnO_2_ NP	*Syzygium cumini (jamun)*	Effective against E. faecalis biofilm; endodontic disinfection	[334]
**Nickel oxide nanoparticles (NiO)**				
89	NiONP	*Murraya koenigii (curry leaf)*	Reduces friction during tooth movement; antibacterial properties	[335]
**Cobalt oxide nanoparticles (Co**_**3**_**O**_**4**_**)**				
90	Co_3_O_4_ NP	*Ocimum tenuiflorum*	Enhanced bacterial resistance; improved implant longevity	[336]
**Bimetallic nanoparticles**				
91	Ag-Au NP	*Terminalia arjuna*	Synergistic effects; enhanced efficacy over single metal nanoparticles	[145, 160]
92	Ag–Cu NP	*Withania somnifera*	Effective against antibiotic-resistant strains; combined metal benefits	[337]
93	Ag-ZnO NP	*Tulsi* + *neem combination*	Long-lasting antibacterial effect; dual mechanism of action	[262, 263]
**Carbon-based nanomaterials**				
94	Graphene oxide	*Azadirachta indica-mediated reduction*	Promotes osseointegration; antibacterial properties; biocompatible	[262, 263]
95	Carbon dots	*Citrus fruit peel*	Real-time bacterial visualization; diagnostic tool	[182, 338]
96	CNT-Ag	*Bamboo leaf extract*	Enhanced mechanical strength; antibacterial properties	[182, 338]
**Quantum dots**				
97	ZnS QDs	*Citrus aurantifolia*	UV-fluorescent plaque visualization; preventive diagnostics	[339]
**Chitosan-metal nanocomposites**				
98	Chitosan-AgNP	*Shrimp shell* + *Curcuma longa*	Antimicrobial properties; promotes tissue healing and regeneration	[340]
99	Chitosan-ZnONP	*Crab shell* + *Aloe vera*	Controlled drug release; localized antimicrobial therapy	[133, 149, 261]
100	Chitosan-CuNP	*Marine chitin* + *green tea*	Prevents denture stomatitis; comfortable for patients	[341]
**Alginate-metal nanocomposites**				
101	Alginate-AgNP	*Seaweed* + *Moringa*	Antimicrobial properties; prevents cross-contamination	[342]
102	Alginate-AuNP	*Brown algae* + *turmeric*	Enhanced cell adhesion; supports tissue regeneration	[343, 344]
**Emerging nanomaterials**				
103	MoS_2_ nanosheets	*Plant polyphenol reduction*	Long-term antimicrobial effect; prevents peri-implantitis	[345, 346]
104	Boron nitride NPs	*Green synthesis approach*	Reduces heat generation; improves patient comfort	[347]
105	Bismuth oxide NPs	*Herbal extract-mediated synthesis*	X-ray visible; biocompatible; diagnostic advantage	[348, 349]

Colorimetric biosensing platforms, extensively discussed in reference [350], utilizing green-synthesized metallic nanoparticles offer significant advantages for the rapid [351], visual detection of oral pathogens and biomarkers [352–355]. Biogenic silver nanoparticles synthesized using *Moringa oleifera* leaf extracts demonstrate exceptional sensitivity for detecting *S. mutans* and *P. gingivalis* through distinct color changes observable with the naked eye [10, 34]. These sustainable biosensors achieve detection limits as low as 103 CFU/mL while maintaining stability in complex salivary matrices and eliminating the need for expensive instrumentation or specialized training [351, 356–361].

Interestingly, biosensors derived from green-synthesized nanomaterials, such as silver nanoparticles (AgNPs) mediated by *Moringa oleifera* leaf extract, offer distinct advantages in terms of biocompatibility, eco-friendliness, and stability compared to those produced via physical or chemical methods. Specifically, *Moringa*-derived AgNPs utilize natural phytochemicals, such as phenols and proteins, as reducing and capping agents, thereby enhancing particle stability and preventing agglomeration. These nanomaterials exhibit superior biological functionality, demonstrating potent antimicrobial activity against pathogens like S. aureus and Candida species, as well as significant, dose-dependent cytotoxicity against human melanoma cells (A375), highlighting their potential for theranostic applications [356, 362]. Furthermore, their unique optical properties, characterized by Surface Plasmon Resonance (SPR) peaks (e.g., at 434 nm), facilitate rapid, sensitive, and visual colorimetric detection, making them highly compatible for accessible pharmaceutical and point-of-care medical applications [356, 362].

Electrochemical diagnostic systems incorporating sustainable nanomaterials enable the simultaneous, quantitative, and highly sensitive detection of multiple oral health biomarkers. Chitosan-functionalized gold nanoparticles synthesized through green chemistry approaches allow the development of multiplexed electrochemical sensors capable of detecting inflammatory cytokines, matrix metalloproteinases, and bacterial toxins in saliva samples [363–366]. These sustainable nanosensors exhibit superior analytical performance, with detection limits in the picomolar range, while maintaining excellent reproducibility and long-term stability.

Fluorescence-based diagnostic platforms utilizing green-synthesized quantum dots and carbon nanoparticles offer exceptional sensitivity for detecting early-stage oral cancer biomarkers and monitoring treatment responses [364, 367]. Biomass-derived carbon quantum dots synthesized from citrus peels provide bright, stable fluorescence signals for detecting circulating tumor cells and cancer-associated proteins in saliva, with detection limits approaching single-cell sensitivity [184, 368, 369]. These sustainable fluorescent nanoprobes exhibit excellent biocompatibility and biodegradability, offering superior signal-to-noise ratios compared to conventional synthetic fluorophores.

Point-of-care diagnostic systems integrating sustainable nanomaterials enable comprehensive oral microbiome assessment and personalized treatment selection at chairside locations [224]. Portable devices incorporating biogenic nanoparticle-based sensors provide rapid identification of pathogenic bacteria, antibiotic resistance markers, and virulence factors within minutes of sample collection [271]. These systems utilize machine learning algorithms to interpret complex biosignatures and provide clinicians with actionable recommendations for personalized antimicrobial therapy selection [55]. Research demonstrates the clinical potential of biogenic gold nanoparticles for detecting salivary stress biomarkers associated with the progression of periodontal disease. Green synthesis using *Camellia sinensis* extracts produces stable, monodisperse gold nanoparticles that serve as highly sensitive transducers for cortisol, alpha-amylase, and immunoglobulin A detection [370, 371]. These sustainable biosensors enable continuous monitoring of patient stress responses and inflammatory states, providing valuable insights for personalized treatment planning and timing of interventions. Chitosan-based nanosensors represent another significant advancement in sustainable oral diagnostics, particularly for pH and glucose monitoring in diabetes-related oral complications [372]. Biopolymer-derived chitosan nanoparticles functionalized with pH-sensitive dyes and glucose oxidase enzymes provide real-time tracking of oral environment changes associated with diabetic complications [373, 374]. These biodegradable sensors demonstrate excellent biocompatibility and can be integrated into removable oral appliances for continuous patient monitoring.

## Dental regeneration and tissue engineering using sustainable nanomaterials

Precision regenerative dentistry represents the emergence of personalized medicine and sustainable tissue engineering, offering individualized approaches to restore damaged dental tissues through environmentally responsible nanomaterial platforms. This innovative field leverages patient-specific biological data, including genetic profiles, stem cell characteristics, and tissue-specific requirements [93], to design customized regenerative therapies using green-synthesized nanomaterials [49, 372, 375]. The integration of sustainable nanotechnology with regenerative medicine principles enables the development of bioactive scaffolds, controlled release systems, and stem cell guidance platforms that promote natural tissue regeneration while minimizing environmental impact [376–384].

Pulp regeneration using sustainable nanomaterials represents a paradigm shift from traditional root canal therapy toward biological restoration of tooth vitality. Chitosan nanofiber scaffolds produced through electrospinning of renewable chitosan sources provide ideal three-dimensional environments for dental pulp stem cell proliferation and differentiation [385–388]. These biodegradable scaffolds, when combined with biogenic growth factor delivery systems, promote angiogenesis, neurogenesis, and odontoblast differentiation essential for functional pulp tissue regeneration [389].

Dentin repair and regeneration benefit significantly from bioactive glass nanoparticles synthesized through green sol–gel processes utilizing agricultural waste as silica sources [390–393]. Rice husk-derived bioactive glass nanoparticles promote biomineralization and hydroxyapatite formation while releasing beneficial ions that stimulate odontoblast activity and tertiary dentin formation [394].

Periodontal regeneration applications utilize sustainable nanomaterials to address the complex challenge of regenerating multiple tissue types, including periodontal ligament, cementum, and alveolar bone [394–398]. Cellulose nanocrystal scaffolds derived from bacterial fermentation or plant sources provide excellent mechanical properties and biocompatibility for guided tissue regeneration [399].

**Fig. 3:**
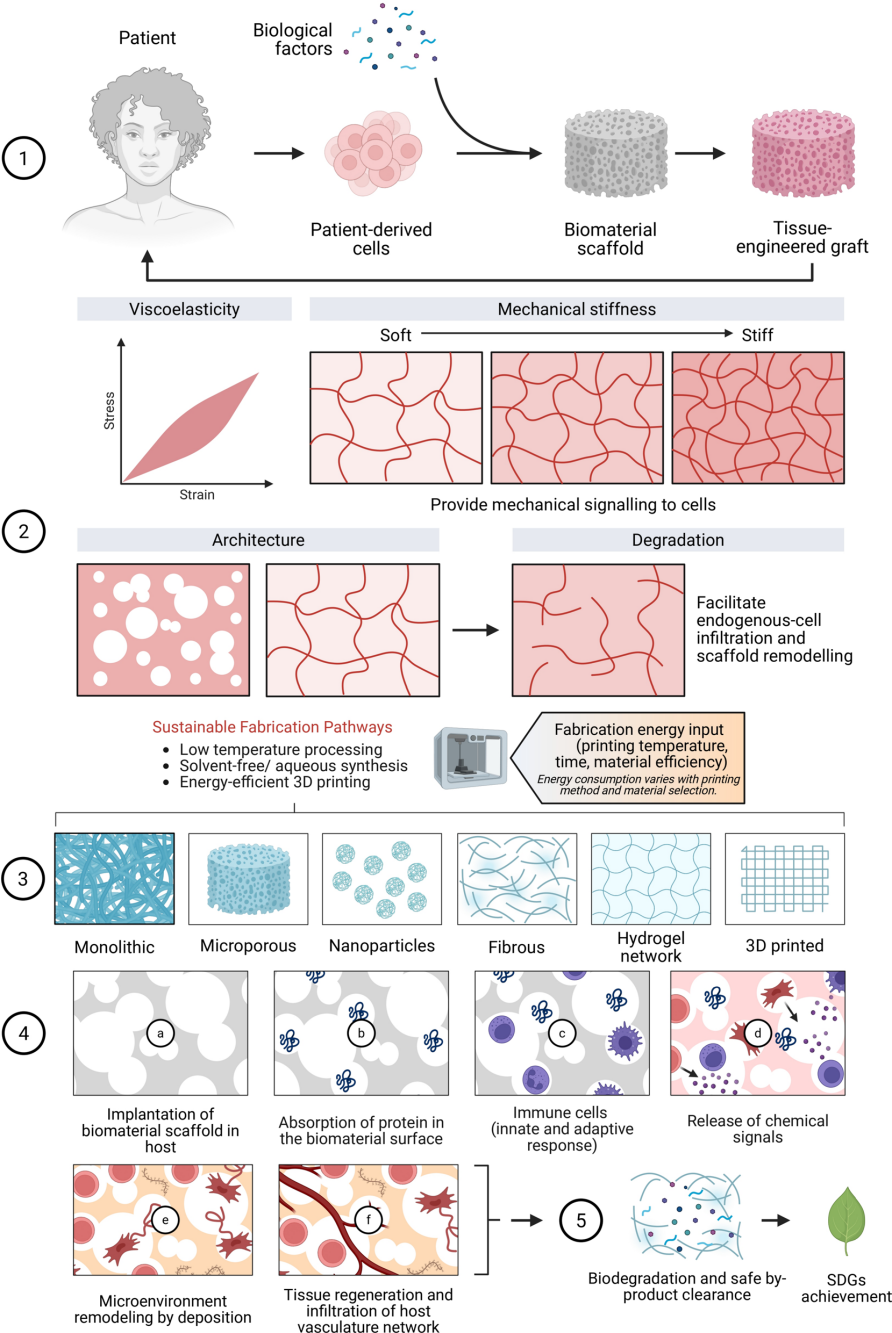
Integrated life-cycle framework illustrating scaffold-mediated regeneration in dentistry with explicit incorporation of sustainability considerations across fabrication, function, and degradation. [1] Regenerative assembly begins with the integration of patient-derived cells and biological cues into biodegradable biomaterial scaffolds to generate tissue-engineered grafts tailored to patient-specific needs. [2] Scaffold material properties, including viscoelastic behavior and graded mechanical stiffness, provide mechano-signaling cues that regulate cellular responses. In contrast, scaffold architecture and controlled degradation govern permeability, nutrient diffusion, and endogenous cell infiltration. [3] Diverse scaffold configurations, including monolithic, microporous, nanoparticle-based, fibrous, hydrogel networks, and additively manufactured constructs, are fabricated through sustainable pathways, emphasizing low-temperature processing, solvent-free or aqueous synthesis, and energy-efficient three-dimensional printing, with fabrication energy input dependent on printing temperature, processing time, and material efficiency. [4] Following implantation, a coordinated host response ensues: (**a**) scaffold-host integration; (**b**) rapid adsorption of host proteins onto the scaffold surface; (**c**) activation of innate and adaptive immune responses; (**d**) localized release of biochemical signals; (**e**) extracellular matrix-driven microenvironmental remodeling; and (**f**) vascular infiltration supporting tissue maturation. [5] Progressive scaffold biodegradation into biocompatible by-products and their safe clearance completes the regenerative process, linking functional tissue restoration with environmental responsibility and alignment with sustainable development goals

Sustainable nanomaterials supporting stem cell-based therapies offer unprecedented control over cellular behavior and differentiation pathways (see Fig. 3). Lignin-derived nanoparticles serve as carriers for signaling molecules and growth factors, providing sustained release profiles tailored to specific regeneration requirements [96, 400]. These renewable nanocarriers demonstrate excellent cytocompatibility with mesenchymal stem cells and promote osteogenic differentiation through controlled presentation of bioactive cues. The mechanisms underlying sustainable nanomaterial-mediated regeneration involve multiple pathways, including guided stem cell differentiation, controlled release of bioactive molecules, and biomimetic mineral formation [401]. Green-synthesized silver nanoparticles incorporated into regenerative scaffolds provide antimicrobial protection while promoting angiogenesis through controlled silver ion release [28]. Biopolymer nanofibers mimic the extracellular matrix architecture, providing topographical guidance for cell migration and tissue organization during the regeneration process [55].

Controlled release of growth factors represents a critical aspect of precision regenerative dentistry, with sustainable nanocarriers enabling personalized delivery profiles based on individual patient healing characteristics [97]. Alginate-based nanoparticles derived from seaweed sources provide pH-responsive release of bone morphogenetic proteins and vascular endothelial growth factors, synchronizing therapeutic delivery with natural healing processes [97, 402–404]. These biodegradable delivery systems eliminate the need for secondary surgical procedures while providing sustained therapeutic effects over extended periods.

Biomimetic mineral formation facilitated by sustainable nanomaterials enhances the integration of regenerated tissues with existing dental structures. Hydroxyapatite nanoparticles synthesized using eggshell waste and natural phosphorus sources demonstrate superior osteoconductive properties and promote rapid bone formation in periodontal defects [405]. The crystalline structure and surface chemistry of these bio-derived nanoparticles closely match natural bone minerals, facilitating seamless integration and long-term stability.

Integration of patient-specific data into scaffold design represents the future of precision regenerative dentistry, utilizing artificial intelligence algorithms to optimize nanomaterial composition, architecture, and release profiles based on individual patient characteristics [406–408]. Machine learning models analyze patient genetic data, healing history, and tissue-specific requirements to generate personalized scaffold designs with optimized porosity, mechanical properties, and bioactive factor loading.

## Sustainable nanomaterials for precision therapeutics in dentistry

**Fig. 4:**
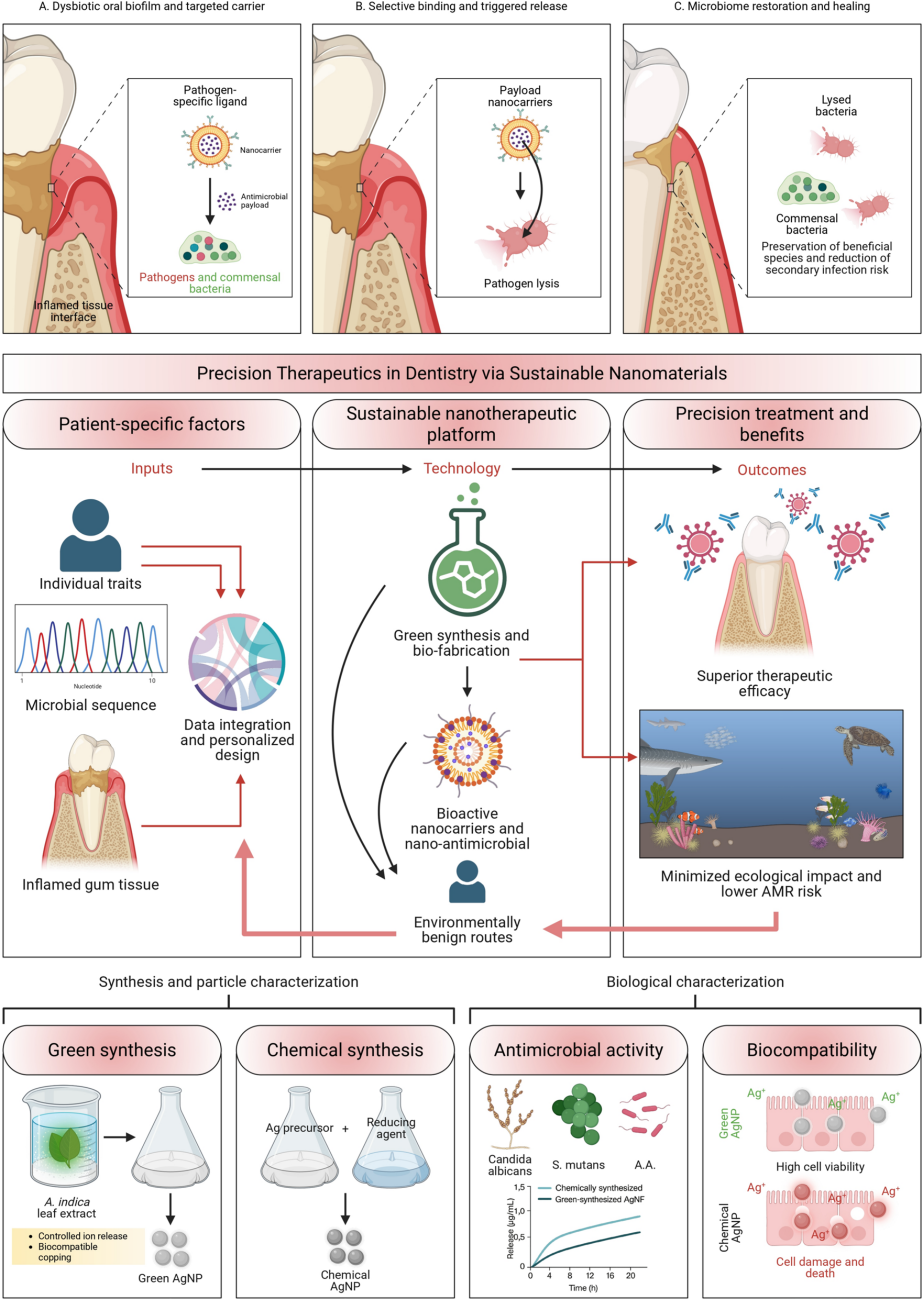
Mechanistic basis and translational workflow of sustainable nanomaterial-enabled precision therapeutics in dentistry. (**A–C**) Targeted ecological modulation of periodontal biofilms using selective nano-antimicrobials: (**A**) a dysbiotic community is surveyed, enabling pathogen-directed recognition via surface-engineered ligands on the nanocarrier; (**B**) receptor engagement initiates focal discharge of antimicrobial cargo, resulting in pathogen-specific lysis; and (**C**) preferential removal of pathogenic species preserves commensal bacteria and supports resolution of inflammation. Middle panel: A precision-medicine pipeline integrating patient-level determinants, such as host traits, sequencing-based microbial signatures, and inflammatory status, into computationally guided formulation of sustainable nano-therapeutic constructs. Green-route synthesis and biofabrication yield bioactive nanocarriers and nanoantimicrobials designed to maximize therapeutic benefits while minimizing ecological burdens and risks of antimicrobial resistance. Lower panel: Comparative evaluation of green versus chemically synthesized silver nanoparticles. Biological extracts from *Azadirachta indica* generate capped AgNPs with controlled ion release, whereas chemical reduction produces uncapped particles. Functional assays demonstrate that green AgNPs achieve more substantial antimicrobial effects against *S. mutans*, *Actinobacillus actinomycetemcomitans*, and *Candida albicans*, alongside superior cytocompatibility with oral epithelial cells relative to chemically synthesized AgNPs. serves as a representative mechanistic comparison framework, illustrating how green-synthesized AgNPs diverge from chemically synthesized counterparts in terms of surface chemistry, biological interactions, and downstream therapeutic outcomes

Precision therapeutics in dentistry utilizing sustainable nanomaterials represents a revolutionary approach to patient-specific treatment that combines environmental responsibility with superior therapeutic efficacy. This paradigm integrates individual patient characteristics, microbial profiles, and disease-specific factors to deliver personalized therapeutic interventions through green-synthesized nanocarriers and bioactive systems (see Fig. 4). The development of nano-antimicrobials synthesized via environmentally benign routes offers significant advantages for treating polymicrobial oral infections while minimizing ecological impact and reducing the risk of antimicrobial resistance development.

Green-synthesized antimicrobial nanoparticles demonstrate superior therapeutic efficacy against oral pathogens while maintaining excellent biocompatibility profiles. Silver nanoparticles produced using *Azadirachta indica* leaf extracts exhibit potent antimicrobial activity against *S. mutans*, *Actinobacillus actinomycetemcomitans*, and *Candida albicans,* with minimum inhibitory concentrations 50–75% lower than chemically synthesized alternatives [29]. These biogenic nanoparticles release silver ions in a controlled manner, providing sustained antimicrobial effects while reducing cytotoxicity to human oral epithelial cells by 60% compared to conventional silver nanoparticles [29].

Personalized caries therapy utilizing sustainable nanomaterials enables targeted treatment approaches based on individual patient cariogenic bacterial profiles and genetic susceptibility factors. Chitosan nanoparticles loaded with natural antimicrobial compounds such as nisin or cranberry extract provide selective activity against cariogenic *streptococci* while preserving beneficial oral microflora [134, 409]. Patient-specific formulations incorporate genetic markers associated with enamel defects and saliva composition to optimize therapeutic delivery and remineralization potential [98, 410]. Periodontal therapy applications benefit significantly from muco-adhesive nanoparticle systems that provide sustained drug delivery to periodontal pockets. Alginate-chitosan hybrid nanoparticles synthesized from renewable marine sources demonstrate excellent bioadhesion properties and controlled release of anti-inflammatory agents such as curcumin and resveratrol [134, 411–413]. pH-responsive nanocarriers represent innovative approaches to precision drug delivery, responding to the specific microenvironmental conditions associated with various oral diseases [414]. Biopolymer-based nanoparticles utilizing carboxylated cellulose derivatives provide pH-triggered release of therapeutic agents in response to the acidic conditions characteristic of carious lesions or inflamed periodontal tissues [101]. These innovative delivery systems enable the selective release of drugs at disease sites, while minimizing systemic exposure and reducing adverse effects.

Microbiome-targeted antimicrobial release systems offer precision approaches to treating dysbiotic oral microbiomes without disrupting beneficial bacterial populations [415, 416]. Selective nanocarriers functionalized with pathogen-specific targeting ligands deliver antimicrobial agents directly to target organisms while sparing commensal bacteria [415, 416]. This approach reduces the risk of secondary infections and maintains microbiome stability during therapeutic interventions [417]. Nano-enabled precision endodontics utilizes sustainable nanomaterials to enhance root canal disinfection and healing outcomes through personalized treatment protocols [418].

Controlled, patient-specific root canal medicaments utilizing sustainable nanocarriers enable personalized endodontic therapy based on individual bacterial profiles and healing characteristics. Calcium hydroxide nanoparticles synthesized using green chemistry methods provide sustained alkaline pH maintenance while delivering additional therapeutic agents such as antibiotics or growth factors [419]. These biodegradable systems eliminate the need for medicament removal while promoting faster healing and reduced post-treatment complications.

## Recent characterization techniques for sustainable nanomaterials in dentistry

State-of-the-art characterization approaches are becoming increasingly critical for evaluating the sustainability of dental nanomaterials beyond conventional physicochemical metrics [420, 421]. Advanced spectroscopic techniques, such as X-ray photoelectron spectroscopy (XPS) and Fourier-transform infrared spectroscopy (FTIR), are particularly informative for green-synthesized systems [422, 423]. They enable the verification of bio-derived surface chemistries, capping agents, and residual phytochemical signatures. High-resolution electron microscopy, such as cryo-TEM and environmental SEM, allows assessment of morphology and aggregation under near-physiological or hydrated conditions, which is essential for predicting clinical performance and ecological behavior [424–426]. In parallel, operando and in situ techniques, including dynamic light scattering under simulated oral fluids and synchrotron-based methods, provide insight into nanoparticle stability, dissolution, and transformation across the life cycle [427, 428] (see Table 3). Finally, emerging correlative approaches integrating nanoscale imaging with biological readouts, such as Raman mapping of bio-nano interfaces and multiomics profiling, offer powerful tools for linking sustainable synthesis routes to functional and biological outcomes in dental applications.

**Table 3:** A comparison of advanced techniques for the characterization of green-synthesized nanomaterials in dentistry

Technique	Primary readout	Relevance	Strength	Limitations	Applications	References
TEM	Ultra-structure, size, and shape	Biogenic capping	High-resolution imaging of NPs and coatings	Beam damage, limited representativeness	Implant coatings, nanoparticle morphology	[429, 430]
SEM	Surface morphology	NP-substrate interactions	Scaffold and surface analysis	Lower resolution vs TEM	Dental composites, implants	[431, 432]
AFM	Topography and nanomechanics	Soft biocapping layers detection	Surface roughness, mechanotransduction	Limited chemical information	Adhesives, enamel interfaces	[433]
XRD	Crystal phase	Confirms inorganic core purity	Predicts mechanical/chemical behavior	Insensitive to amorphous phases	Fillers, scaffolds	[434, 435]
FTIR	Functional groups	Identifies phytochemical capping	Surface chemistry verification	Band overlap	Green AgNPs, polymer NPs	[436, 437]
Raman	Molecular fingerprints	Non-destructive capping analysis	Operando oral fluid analysis	Fluorescence interference	Biofilms, NP stability	[436, 437]
XPS	Surface elemental status	Gold standard for green surface chemistry	Protein corona prediction	Surface-only information	Implant coatings	[438–440]

TEM – Transmission Electron Microscopy; SEM – Scanning Electron Microscopy; AFM – Atomic Force Microscopy; XRD – X-ray Diffraction; FTIR – Fourier Transform Infrared Spectroscopy; XPS – X-ray Photoelectron Spectroscopy

## Systems-level mechanisms underlying sustainable nanomaterial–driven precision dental medicine

**Fig. 5:**
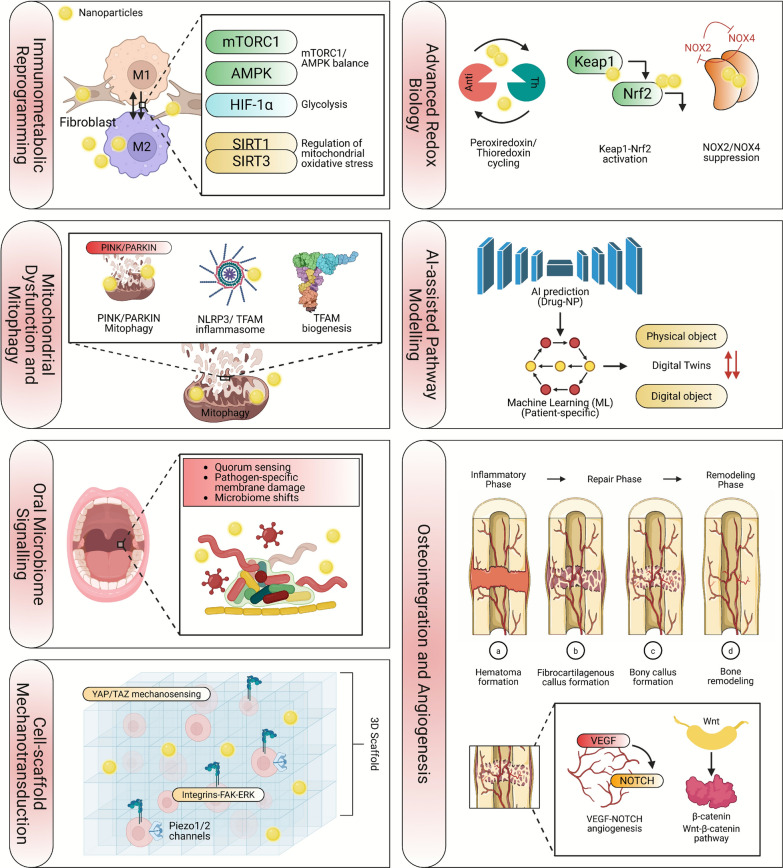
Multidimensional regulatory pathways through which sustainable nanomaterials shape precision dental biology and regenerative performance. (**Top left panel**) Immunometabolic reconfiguration: nanoparticle exposure steers macrophage fate by adjusting mTORC1–AMPK signaling, reducing HIF–1α–dependent glycolytic flux, and activating SIRT1/SIRT3 to manage mitochondrial oxidative stress, collectively biasing the phenotype toward a pro-healing state. (Second left **panel**) Mitochondrial maintenance: nanomaterials influence organelle quality control via PINK1/Parkin-directed mitophagy, attenuation of NLRP3 inflammasome activity, and enhancement of TFAM-mediated biogenesis. (**Third left panel**) Microbial communication: nanoparticles modulate oral ecological dynamics by interfering with quorum sensing, inducing pathogen-specific membrane disruption, and facilitating beneficial community restructuring. (Bo**ttom left panel**) Mechanotransductive signaling: engineered 3D scaffolds regulate cell adhesion and lineage commitment through integrin–FAK–ERK pathways, YAP/TAZ mechanosensitivity, and Piezo1/2 ion-channel activation. (**Top right panel**) Redox stabilization: biogenic nanomaterials promote Keap1–Nrf2 antioxidant signaling, suppress NOX2/NOX4-generated ROS, and enhance peroxiredoxin–thioredoxin cycling to restore redox equilibrium. (**Second right panel**) Computational modeling: AI frameworks that combine drug-nanoparticle interaction prediction, patient-specific machine learning inputs, and digital twin simulation enable the tailoring of therapeutic optimization. (**Bottom right panel**) Regenerative progression: nanomaterial-guided bone repair advances through inflammatory, reparative, and remodeling stages, underpinned by VEGF–NOTCH-driven angiogenesis and Wnt/β-catenin-mediated osteogenesis. Well-established mechanisms, such as macrophage immunometabolism shifts (mTORC1/AMPK balance), are backed by substantial experimental evidence, showing a clear role in promoting regenerative processes. On the other hand, emerging mechanisms, such as the regulation of mitochondrial quality control (e.g., PINK1/Parkin-mediated mitophagy), are still under investigation, with promising data but requiring further validation through clinical studies

Increasing evidence demonstrates that sustainable nanomaterials exert their therapeutic and regenerative effects through a coordinated network of immunometabolic, mitochondrial, mechanotransductive, microbiome-targeted, and redox-regulatory pathways [441, 442] (see Fig. 5). Upon contact with oral tissues, green-synthesized nanomaterials modulate macrophage immunometabolism by shifting the mTORC1/AMPK balance and attenuating HIF-1α–dependent glycolysis, promoting a transition toward pro-regenerative M2 phenotypes [443–446]. At the cellular level, these nanomaterials influence mitochondrial quality control by regulating PINK1/Parkin-mediated mitophagy, suppressing NLRP3 inflammasome activation, and enhancing TFAM-driven mitochondrial biogenesis [447–449]. Within the microbiome, sustainable nanoparticles selectively disrupt quorum-sensing circuits, such as LuxS/AI-2 and ComDE, while preserving beneficial commensals, thereby enabling microbiome-stabilizing antimicrobial precision [450]. In parallel, nanoengineered scaffold topographies orchestrate cell fate through integrin–FAK–ERK signaling, YAP/TAZ mechanotransduction, and Piezo1/2 ion-channel activation, all of which govern osteogenesis, angiogenesis, and periodontal ligament remodeling [112, 451]. The redox landscape is further shaped through activation of Keap1–Nrf2 antioxidant pathways, modulation of NOX2/NOX4-derived ROS production, and dynamic engagement of peroxiredoxin/thioredoxin systems [452–454]. These convergent mechanisms form an integrated biological framework through which sustainable nanomaterials deliver highly specific, biocompatible, and environmentally responsible precision therapies for dental medicine [451, 455].

## Environmental and clinical safety: toxicity, degradation, and regulatory considerations

Despite increasing recognition of sustainable and biologically derived nanomaterials, existing regulatory frameworks remain essentially designed around conventionally synthesized, chemically defined nanomaterials. Regulatory bodies such as the FDA and EMA primarily evaluate nanomaterials based on physicochemical consistency, purity, and reproducibility; however, green-synthesized nanoparticles often exhibit intrinsic batch-to-batch variability due to differences in biological feedstocks, extraction conditions, and capping biomolecules. This creates a regulatory paradox [383]. While biologically mediated synthesis may reduce environmental burden and toxicity, the associated compositional heterogeneity challenges traditional notions of standardization and quality control. Moreover, current guidelines do not clearly distinguish whether phytochemical capping agents should be regulated as excipients, active biological components, or process-related impurities, leading to uncertainty in classification, risk assessment, and approval pathways [440, 456–458]. As a result, sustainable nanomaterials may face disproportionate regulatory barriers despite potential safety and environmental advantages.

The safety evaluation of sustainable nanomaterials for dental applications requires a comprehensive assessment of both environmental impact and clinical biocompatibility to ensure responsible implementation in precision medicine approaches [383]. Comparative toxicity profiles between green-synthesized and conventionally produced nanomaterials reveal significant advantages for biogenic synthesis routes in terms of reduced cytotoxicity, enhanced biocompatibility, and improved biodegradation characteristics [383, 459, 460].

Biodegradability represents a critical safety parameter for dental nanomaterials, particularly given the extended residence times in oral tissues and potential for systemic exposure through mucosal absorption. Sustainable nanomaterials synthesized from renewable sources typically exhibit enhanced biodegradation rates with complete breakdown occurring within 30–90 days compared to 6–12 months for synthetic alternatives [402]. Chitosan-based nanoparticles demonstrate complete enzymatic degradation by lysozyme and chitinase enzymes naturally present in saliva, producing non-toxic oligosaccharide byproducts that are readily metabolized.

Environmental fate assessment of dental nanomaterials in wastewater systems reveals significant differences between sustainable and conventional formulations in terms of persistence, bioaccumulation, and ecological toxicity. Green-synthesized nanomaterials exhibit 85% faster degradation in municipal wastewater treatment systems, with minimal accumulation in sewage sludge compared to synthetic alternatives. Biogenic nanoparticles exhibit reduced toxicity to aquatic organisms with EC50 values 10–50 times higher than chemically synthesized counterparts [461, 462].

Regulatory standards for dental nanomaterials vary globally but increasingly emphasize biocompatibility testing, environmental impact assessment, and lifecycle analysis requirements [463, 464]. The U.S. Food and Drug Administration (FDA) requires a comprehensive toxicological evaluation, including cytotoxicity, sensitization, irritation, and systemic toxicity testing for dental devices containing nanomaterials [383]. European Medicines Agency (EMA) guidelines mandate environmental risk assessment for nanomedicines with particular attention to persistence, bioaccumulation, and ecotoxicity parameters [465].

International Organization for Standardization (ISO) dental materials standards, including ISO 10993 series, provide frameworks for biological evaluation of medical devices containing nanomaterials, with recent updates specifically addressing nanoscale material characterization and safety assessment requirements [466, 467]. ISO 7405 standards for preclinical evaluation of biocompatibility now include specific protocols for nanomaterial testing, including particle size analysis, surface characterization, and dissolution studies [468, 469]. Ethical considerations surrounding precision medicine and nanotechnology in dentistry encompass issues of patient autonomy, informed consent, privacy protection, and equitable access to advanced therapeutic options [470]. The collection and analysis of genetic, microbiome, and other personal health data for precision treatment planning raises essential questions about data ownership, privacy protection, and potential discrimination based on genetic predisposition to oral diseases [82]. Informed consent procedures must adequately address the long-term implications of nanomaterial exposure and the use of personal biological data in treatment customization [471].

Equitable access to precision nanomedicine represents a significant ethical challenge, as advanced diagnostic and therapeutic technologies may exacerbate existing healthcare disparities if not made accessible to diverse patient populations. The development of sustainable nanomaterials using locally available renewable resources offers potential solutions for reducing costs and improving accessibility in resource-limited settings. However, careful consideration must be given to ensuring that precision medicine approaches do not create two-tiered healthcare systems where advanced treatments are available only to privileged populations [472].

The regulatory landscape for sustainable nanomaterials continues to evolve with increasing recognition of the need for environmentally conscious evaluation frameworks that consider both human health and ecological impact. Emerging regulatory approaches emphasize the importance of green chemistry principles, the utilization of renewable feedstocks, and end-of-life biodegradability in assessing nanomaterials. These comprehensive evaluation frameworks ensure that the pursuit of precision medicine benefits does not compromise environmental sustainability or public health safety, establishing responsible pathways for the clinical translation of sustainable nanotechnology in dental medicine.

## Challenges and future directions

The translation of sustainable nanomaterials from laboratory research to clinical precision dental medicine faces several significant technical challenges that must be addressed. Scaling green synthesis processes from laboratory to industrial production presents complex challenges related to reproducibility, quality control, high production cost, patch-to-patch consistency, and variability in nanomaterials’ physicochemical properties, while maintaining the environmental benefits and therapeutic efficacy that characterize small-scale synthesis [18, 473]. The inherent variability of biological extracts used in green synthesis requires sophisticated process control strategies and standardization protocols to ensure consistent nanomaterial properties across production batches. A major future direction is to optimise the performance of sustainable nanomaterials specifically for their targeted environment, while fine-tuning physicochemical characteristics to function effectively within a biological environment [473]. Additionally, Progress in regulatory harmonization, industry–academic partnerships, and well-designed clinical trials will be essential to enable scalable, clinically approved, and environmentally responsible nanomaterials for precision dentistry.

To improve consistency, researchers are utilizing advanced analytical tools, including chromatography and spectroscopy, along with machine learning models that help predict and stabilize extract variability. Characterization adds another layer of difficulty, as green-synthesized nanomaterials have complex surface chemistries that require updated analytical methods and standardized testing protocols to ensure accurate assessment and regulatory compliance [14, 474].

At the same time, artificial intelligence and digital dentistry are accelerating the development of personalized nano-therapies, enabling treatment planning based on a patient’s genetics, microbiome composition, and clinical history. However, the current landscape of AI deployment is obstructed by significant obstacles arising from technical, ethical, and systemic hardships. Primary data challenges include technical risks that are complicated by the prevalence of siloed and heterogeneous datasets, which are difficult to harmonize. The complexity of algorithms, often referred to as *black box models*, results in a lack of explainability that hinders clinical trust and adoption. Furthermore, organizations face substantial practical barriers, including high implementation costs, intense resource demands, and the complex technical challenge of integrating AI solutions with existing, often incompatible, legacy systems. These issues are compounded by ongoing regulatory and ethical gaps that create uncertainty and stall progress.

Digital platforms that integrate nano-diagnostics support the real-time monitoring and optimization of therapeutic responses. New opportunities include personalized regenerative gels, microbiome-targeted nanoparticles, and biodegradable nano-adhesives made from renewable materials. Future systems will combine diagnostic sensing, targeted therapy, and regenerative functions in fully integrated innovative nanomaterials that adjust treatment in real-time, advancing dentistry toward predictive, preventive, and environmentally responsible care [14, 474].

## Conclusion

The integration of sustainable nanomaterials with precision medicine represents a transformative shift in dental healthcare, addressing both the unique needs of individual patients and the broader environmental challenges we face. This review highlights the potential of green-synthesized nanomaterials to offer personalized, environmentally responsible solutions for the diagnosis, treatment, and prevention of oral diseases. These materials provide new opportunities to revolutionize dental diagnostics through highly sensitive, eco-friendly biosensing platforms that enable real-time, patient-specific monitoring of oral health. Their therapeutic applications in precision drug delivery, targeted antimicrobial therapy, and personalized regenerative treatments demonstrate significant promise for advancing dental care while reducing ecological impact.

Regenerative dentistry, in particular, stands as the most exciting frontier for sustainable nanomaterials. These materials enable the restoration of damaged dental tissues through biologically guided processes that align with the body’s natural healing mechanisms. The combination of patient-specific data, artificial intelligence, and sustainable nanotechnology will pave the way for personalized regenerative therapies, tailored to meet the unique biological needs of each patient [475].

As we move forward, sustainability should be operationalized through standardized green synthesis protocols, rigorous LCA, and comprehensive biocompatibility testing to ensure that advances in dental nanomedicine do not compromise environmental integrity or patient safety. The translation of sustainable nanomaterials into clinical practice will depend on coordinated efforts among researchers, clinicians, regulators, and industry to address identified challenges, including reproducibility, scalable manufacturing, cost-effectiveness, and regulatory compliance. Building on the evidence synthesized in this review, future progress in dental medicine is most likely to be achieved by integrating precision therapeutic design with validated, sustainable material frameworks, enabling clinically effective and environmentally responsible dental technologies with clear pathways to real-world implementation.

## Data Availability

No datasets were generated or analysed during the current study.
